# Treatment outcomes of trastuzumab-based chemotherapy in patients with HER2-positive gastric cancer: a nationwide retrospective cohort study

**DOI:** 10.1093/oncolo/oyag196

**Published:** 2026-05-20

**Authors:** Tae-Hwan Kim, Eunyoung Lee, Yong Won Choi, Mi Sun Ahn, Seok Yun Kang, Jin-Hyuk Choi, Hyun Woo Lee

**Affiliations:** Department of Hematology-Oncology, Ajou University School of Medicine, Suwon 16499, Korea; Department of Neurology, McGovern Medical School, University of Texas Health Science Center at Houston, Houston, TX 77030, United States; Department of Hematology-Oncology, Ajou University School of Medicine, Suwon 16499, Korea; Department of Hematology-Oncology, Ajou University School of Medicine, Suwon 16499, Korea; Department of Hematology-Oncology, Ajou University School of Medicine, Suwon 16499, Korea; Department of Hematology-Oncology, Ajou University School of Medicine, Suwon 16499, Korea; Department of Hematology-Oncology, Ajou University School of Medicine, Suwon 16499, Korea

**Keywords:** outcomes, trastuzumab, HER2, gastric cancer, nationwide

## Abstract

**Background:**

Trastuzumab-based chemotherapy is the standard first-line treatment for patients with human epidermal growth factor receptor 2 (HER2)–positive advanced or metastatic gastric cancer (GC). However, evidence on real-world outcomes according to the chemotherapy backbone remains limited.

**Methods:**

A total of 2399 patients with GC treated with trastuzumab-based chemotherapy were analyzed, of whom 486 patients were treated with trastuzumab/5-fluorouracil/cisplatin (HFP) and 1913 patients were treated with trastuzumab/capecitabine/cisplatin (HXP). Time to subsequent treatment (TST) and overall survival (OS) were analyzed according to the type of chemotherapy backbone.

**Results:**

With a median follow-up of 10.5 months, the median TST was 6.08 months (95% confidence interval [CI], 5.65–6.60) in the HFP group and 8.02 months (95% CI, 7.72-8.57) in the HXP group (*P *< .0001). The median OS was 9.89 months (95% CI, 8.94-10.87) for the HFP group and 13.27 months (95% CI, 12.75-14.00) for the HXP group (*P *< .0001). Specifically, HXP followed by trastuzumab and capecitabine maintenance (HXP-HX) demonstrated the best outcomes with median TST of 13.44 months (95% CI, 12.52-15.61) and median OS of 22.18 months (95% CI, 20.83-24.44) compared with HXP followed by trastuzumab maintenance (HXP-H) and HXP alone (both *P *< .0001), which were also significant in multivariable analysis.

**Conclusions:**

Trastuzumab/capecitabine/cisplatin followed by trastuzumab/capecitabine maintenance showed the best survival outcomes for patients with locally advanced unresectable or metastatic HER2-positive GC based on real-world big data analysis of the Trastuzumab for Gastric Cancer regimen, suggesting that HXP-HX is recommended over HXP-H. In the current immunotherapy era for HER2-positive GC, the potential role of capecitabine maintenance as part of combination strategies should be interpreted cautiously and warrants further investigation.

Implications for Practice In patients with human epidermal growth factor receptor 2 (HER2)–positive advanced or metastatic gastric cancer (GC), trastuzumab plus capecitabine and cisplatin (HXP) followed by maintenance with trastuzumab and capecitabine demonstrated the most favorable survival outcomes in real-world practice. These findings support the continuation of both trastuzumab and capecitabine as maintenance therapy after initial HXP treatment. In the immunotherapy era for HER2-positve GC, the potential role of capecitabine maintenance in combination strategies warrants further investigation to determine its impact on clinical benefit and survival.

## Introduction

Gastric cancer (GC) is one of the most common newly diagnosed malignancies in Korea and the fourth most common malignancy worldwide.^1–3^ Despite advances in diagnosis and treatment, GC remains one of the leading causes of cancer-related mortality worldwide, particularly in East Asian countries.^4^^,^^5^ Human epidermal growth factor receptor 2 (HER2)-positive GC accounts for 10%-20% of all GCs,^6^^,^^7^ and trastuzumab is a monoclonal antibody targeting the HER2 receptor, which inhibits tumor cell proliferation and mediates antibody-dependent cellular cytotoxicity, thereby improving survival outcomes in HER2-positive GC.^8^^,^^9^ Trastuzumab-based chemotherapy has become the standard first-line treatment according to the results of the Trastuzumab for Gastric Cancer (ToGA) trial in patients with locally advanced unresectable or metastatic HER2-positive GC.^8^

In the ToGA trial, the chemotherapy backbone with capecitabine/cisplatin or 5-fluorouracil (5-FU)/cisplatin was used for a maximum of 6 cycles.^8^ Several studies have explored chemotherapy backbones, including those with capecitabine maintenance beyond 6 cycles or substitution of cisplatin with oxaliplatin.^10–13^ However, few studies have analyzed treatment outcomes based on the chemotherapy backbone in real-world practice using the ToGA regimen.^12^^,^^13^

This study aimed to analyze real-world big data from the Korean Health Insurance Review and Assessment Service (HIRA) to investigate the potential differences in treatment outcomes according to the chemotherapy backbone of the ToGA regimen.

## Methods

### Patients

**Figure 1.**
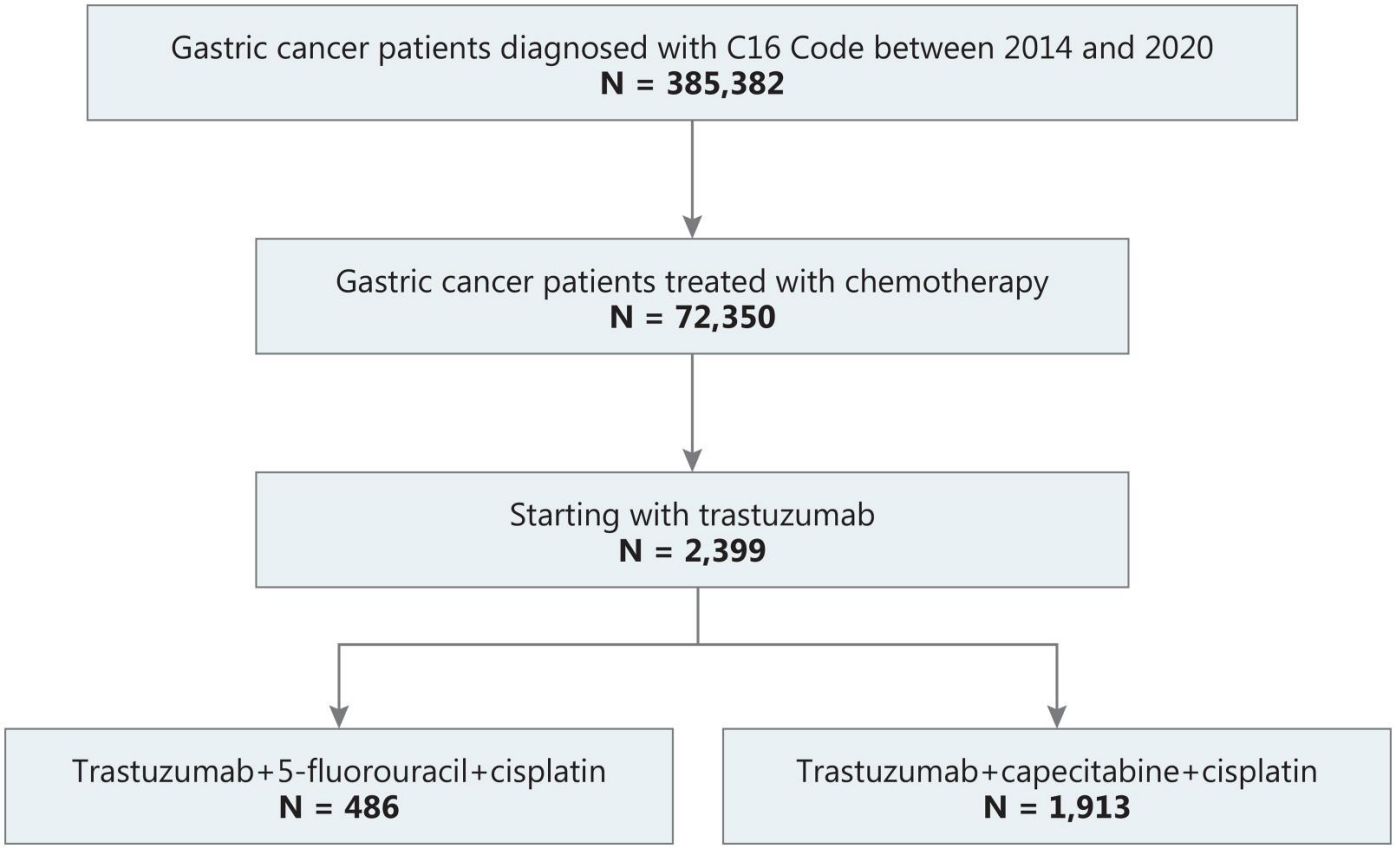
Flowchart of patient selection for the study. The C16 code denotes gastric cancer according to the ICD-10 classification.

A total of 385 382 patients diagnosed with GC with C16 code (the International Classification of Diseases-10) were identified from January 2014 to December 2020 in the Korean HIRA database.^14^ Among them, 72 350 patients who started treatment with any type of chemotherapeutic agents were identified. Among them, 2399 patients who used trastuzumab, a reimbursable drug for locally advanced unresectable or metastatic HER2-positive GC in the Korean health insurance system, were identified. The study population consisted of 486 patients treated with trastuzumab/5-fluorouracil/cisplatin (HFP) and 1913 patients treated with trastuzumab/capecitabine/cisplatin (HXP), all of whom were included in the final analysis (Figure 1).

### Clinical review and definition of survival outcomes

Baseline characteristics were compared between the HFP and HXP groups based on age, sex, the number of trastuzumab administrations, diabetes mellitus (DM), hypertension (HTN), coronary artery occlusive disease (CAOD), chronic kidney disease (CKD), liver cirrhosis (LC), chronic obstructive pulmonary disease (COPD), and history of cancer diagnosis.

Due to the inability to identify the exact dates of disease progression from the HIRA data, subsequent treatment was identified as the date of prescribing other chemotherapeutic agents. Additionally, since the exact date of patient death could not be confirmed in the HIRA data, death was defined as the absence of clinical records or drug prescriptions for more than 6 months, with the date of death defined as the date of the patient’s last medical record.^15^

Time to subsequent treatment (TST) was defined as the period from the initiation of trastuzumab treatment to the start of other chemotherapeutic agents or death, and overall survival (OS) was defined as the period from the start of trastuzumab treatment to death.

### Statistical analysis

Baseline characteristics were compared between chemotherapeutic regimen groups using the chi-square test for categorical variables and the *t*-test for continuous variables. The median TST and OS were calculated using the Kaplan-Meier method with 6- and 12-month survival rates estimated from these curves. Survival outcomes between different chemotherapeutic regimens were compared using the log-rank test. The proportional hazards assumption was verified using Schoenfeld residuals before conducting Cox proportional hazard regression analyses. Both univariable and multivariable Cox proportional hazards models were used to analyze the associations between survival outcomes and the chemotherapeutic regimens. The multivariable analysis was adjusted for potential confounding factors, including patients’ age, sex, DM, HTN, CAOD, CKD, LC, COPD, and history of cancer. All statistical analyses were two-sided and performed using the SAS software (version 9.4; SAS Institute).

## Results

### Patient characteristics

A total of 2399 patients were analyzed, comprising 486 in the HFP group and 1913 in the HXP group. The HFP group included 410 patients (84.4%) treated with HFP alone and 76 patients (15.6%) treated with HFP followed by trastuzumab maintenance (HFP-H). In the HXP group, 1177 patients (61.5%) were treated with HXP alone, 308 (16.1%) with HXP followed by trastuzumab maintenance (HXP-H), and 428 (22.4%) with HXP followed by trastuzumab and capecitabine maintenance (HXP-HX). Patient characteristics are summarized in Table 1. The mean age was 61.83 years, with a statistically higher proportion of patients aged 70 years or older in the HFP group. Male patients were predominant in both groups, and there were no significant differences in comorbidities between the two groups, except for HTN and CKD. Additionally, the number of patients treated with more than 7 cycles of trastuzumab was not significantly different between the two groups.

**Table 1:** Patients’ characteristics.

Characteristics	All	HFP	HXP	*P*
*N* (%)	2399	486	1913	-
		HFP-H: 76 (15.64)	HXP-H: 308 (16.10)	
		HFP: 410 (84.36)	HXP-HX: 428 (22.37)	
			HXP: 1177 (61.53)	
**Median follow-up duration, months [IQR]**	10.51 [5.36, 18.69]	8.43 [4.14, 13.96]	11.40 [5.82, 19.68]	<.0001
**Age, years, mean (SD)**	61.83 (11.53)	62.26 (12.74)	61.72 (11.21)	.3969
**Age ≥70 years, *N* (%)**	654 (27.26)	153 (31.48)	501 (26.19)	.0193
**Sex, male, *N* (%)**	1903 (79.32)	374 (76.95)	1529 (79.93)	.1485
**Trastuzumab prescription ≥7, *N* (%)**	631 (26.3)	128 (26.34)	503 (26.29)	.9844
**No. of trastuzumab prescriptions, median [IQR]**		5 [2, 7]	6 [3, 7]	–
**DM, *N* (%)**	437 (18.22)	100 (20.58)	337 (17.62)	.1311
**HTN, *N* (%)**	703 (29.30)	163 (33.54)	540 (28.23)	.0216
**CAOD, *N* (%)**	23 (0.96)	5 (1.03)	18 (0.94)	.7971
**CKD, *N* (%)**	19 (0.79)	9 (1.85)	10 (0.52)	.007
**LC, *N* (%)**	6 (0.25)	1 (0.21)	5 (0.26)	–
**COPD, *N* (%)**	48 (2.00)	10 (2.06)	38 (1.99)	.9203
**History of cancer, *N* (%)**	1012 (42.18)	197 (40.53)	815 (42.60)	.4097

Abbreviations: CAOD, chronic ischemic heart disease (ICD-10: I25); CKD, chronic kidney disease (ICD-10: N18); COPD, chronic obstructive pulmonary disease (ICD-10: J43-J44); DM, diabetes mellitus (International Classification of Diseases, Tenth Revision (ICD-10): E10-E14); HFP, trastuzumab/fluorouracil/cisplatin; HFP-H, trastuzumab/fluorouracil/cisplatin followed by trastuzumab maintenance; HTN, hypertension (ICD-10: I10-I15); HXP, trastuzumab/capecitabine/cisplatin; HXP-H, trastuzumab/capecitabine/cisplatin followed by trastuzumab maintenance; HXP-HX, trastuzumab/capecitabine/cisplatin followed by trastuzumab/capecitabine maintenance; IQR, interquartile range; LC, cirrhosis of liver (ICD-10: K746); history of cancer (ICD-10: C-code except C16, including patients with C-codes for accompanying metastatic sites).

### Patient outcomes

**Figure 2.**
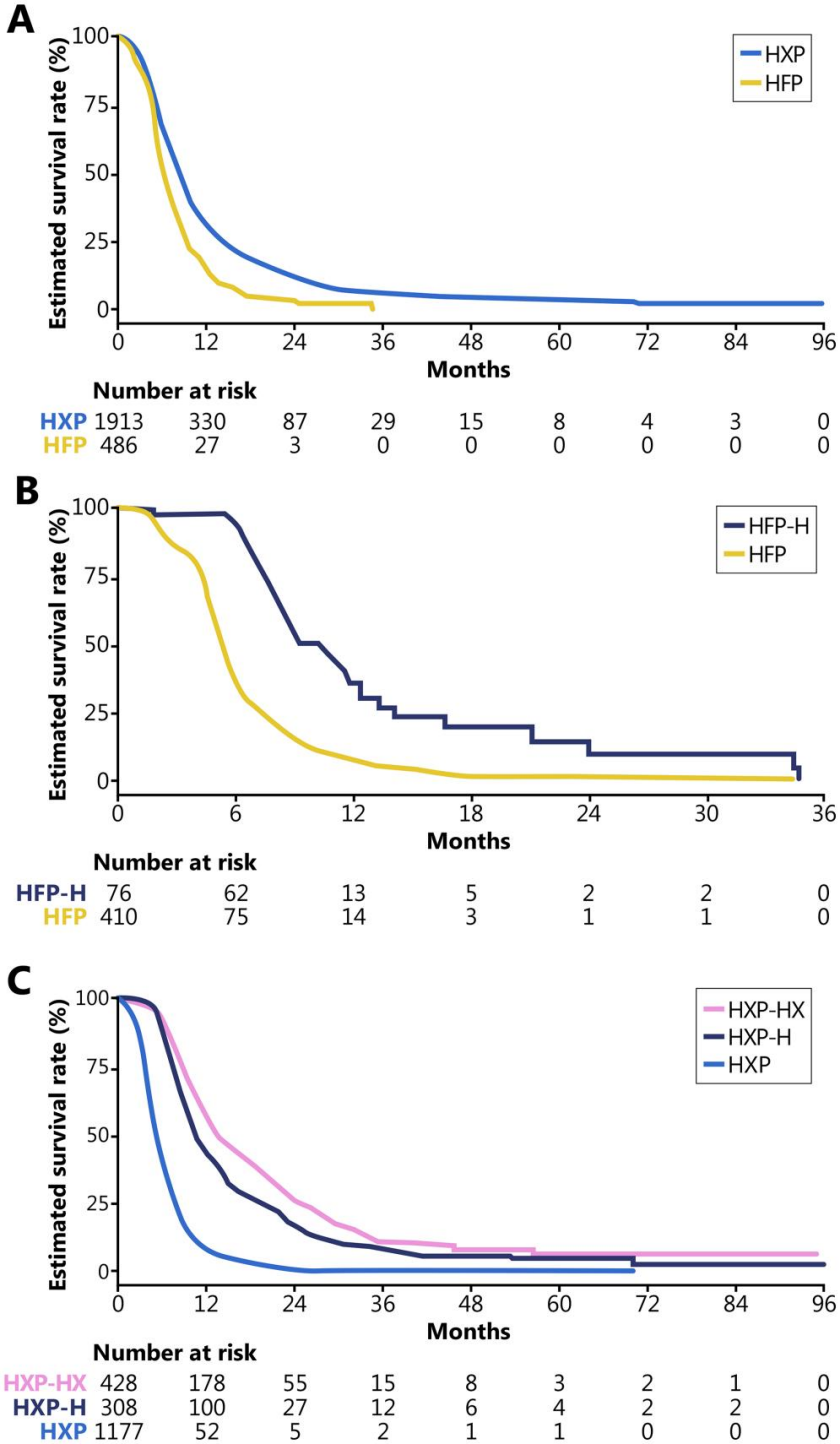
Kaplan-Meier curves showing time to subsequent treatment among trastuzumab-based chemotherapy groups: (A) HFP vs HXP, (B) HFP-H vs HFP alone, and (C) HXP-HX vs HXP-H vs HXP alone. HFP, trastuzumab, fluorouracil, and cisplatin; HXP, trastuzumab, capecitabine, and cisplatin; HFP-H, trastuzumab, fluorouracil, and cisplatin followed by trastuzumab maintenance; HXP-HX, trastuzumab, capecitabine, and cisplatin followed by maintenance with trastuzumab and capecitabine; HXP-H, trastuzumab, capecitabine, and cisplatin followed by trastuzumab maintenance.

With a median follow-up duration of 10.5 months, the median TST was 6.08 months (95% confidence interval [CI], 5.65-6.60) in the HFP group and 8.02 months (95% CI, 7.72-8.57) in the HXP group (*P *< .0001, Table 2, Figure 2A). In the HFP group, patients treated with HFP-H had a median TST of 10.15 months (95% CI, 8.35-11.70), which was significantly longer than the 5.36 months (95% CI, 4.99-5.55) observed in those treated with HFP alone (*P *< .0001, Table 2, Figure 2B). In the HXP group, median TST was 13.44 months (95% CI, 12.35–18.53) for those treated with HXP-HX, 10.64 months (95% CI, 9.63-12.25) for those treated with HXP-H, and 5.03 months (95% CI, 4.93-5.42) for those treated with HXP alone (*P *< .0001, Table 2, Figure 2C).

**Table 2:** Time to subsequent treatment according to the chemotherapeutic regimen.

Regimen	Median TST (95% CI) months	Log-rank *P* value	6-month subsequent-treatment-free rate (95% CI)	12-month subsequent-treatment-free rate (95% CI)
**HFP**	6.08 (5.65-6.60)	<.0001	0.51 (0.45-0.57)	0.14 (0.10-0.18)
**HXP**	8.02 (7.72-8.57)		0.66 (0.64-0.69)	0.31 (0.28-0.33)
**HFP-H**	10.15 (8.35-11.70)	<.0001	0.93 (0.84-0.97)	0.36 (0.23-0.49)
**HFP alone**	5.36 (4.99-5.55)		0.37 (0.31-0.44)	0.07 (0.04-0.11)
**HXP-HX**	13.44 (12.52-15.61)	<.0001	0.93 (0.90-0.95)	0.57 (0.52-0.62)
**HXP-H**	10.64 (9.63-12.25)		0.90 (0.86-0.93)	0.45 (0.38-0.51)
**HXP alone**	5.03 (4.93-5.42)		0.40 (0.37-0.44)	0.09 (0.07-0.11)

Abbreviations: HFP, trastuzumab/5-fluorouracil/cisplatin; HFP-H, trastuzumab/5-fluorouracil/cisplatin followed by trastuzumab maintenance; HXP, trastuzumab/capecitabine/cisplatin; HXP-H, trastuzumab/capecitabine/cisplatin followed by trastuzumab maintenance; HXP-HX, trastuzumab/capecitabine/cisplatin followed by trastuzumab/capecitabine maintenance; TST, time to subsequent treatment.

**Figure 3.**
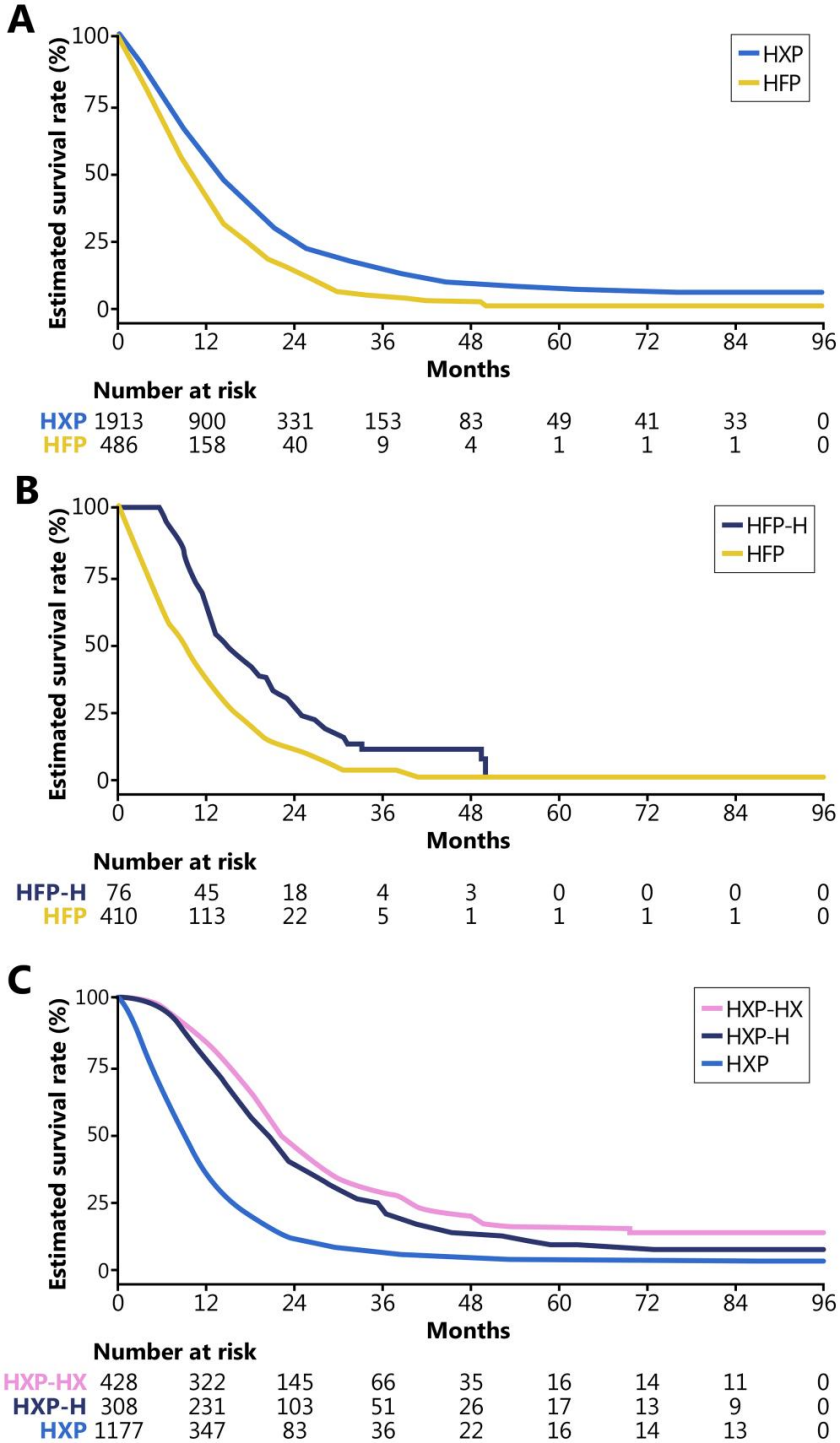
Kaplan-Meier overall survival curves comparing trastuzumab-based chemotherapy regimens: (A) HFP vs HXP, (B) HFP-H vs HFP alone, and (C) HXP-HX vs HXP-H vs HXP alone. HFP, trastuzumab, fluorouracil, and cisplatin; HXP, trastuzumab, capecitabine, and cisplatin; HFP-H, trastuzumab, fluorouracil, and cisplatin followed by trastuzumab maintenance; HXP-HX, trastuzumab, capecitabine, and cisplatin followed by maintenance with trastuzumab and capecitabine; HXP-H, trastuzumab, capecitabine, and cisplatin followed by trastuzumab maintenance.

The median OS for HFP and HXP showed a significant difference, with 9.89 months (95% CI, 8.94-10.87) in the HFP group and 13.27 months (95% CI, 12.75-14.00) in the HXP group (*P *< .0001, Table 3, Figure 3A). In the HFP group, the median OS was 15.15 months (95% CI, 12.35–18.53) for those treated with HFP-H and 8.90 months (95% CI, 7.82-9.89) for those treated with HFP alone (*P *< .0001, Table 3, Figure 3B). Additionally, in the HXP group, the median OS was 22.18 months (95% CI, 20.83-24.44) for those treated with HXP-HX, 20.14 months (95% CI, 18.00-22.31) for those treated with HXP-H, and 9.10 months (95% CI, 8.35-9.66) for those treated with HXP alone (*P *< .0001, Table 3, Figure 3C).

**Table 3:** Overall survival according to the chemotherapeutic regimen.

Regimen	Median OS (95% CI) months	Log-rank *P* value	6-month OS rate (95% CI)	12-month OS rate (95% CI)
**HFP**	9.89 (8.94-10.87)	<.0001	0.68 (0.63-0.72)	0.41 (0.37-0.46)
**HXP**	13.27 (12.75-14.00)		0.79 (0.77-0.81)	0.55 (0.53-0.58)
**HFP-H**	15.15 (12.35-18.53)	<.0001	0.96 (0.88-0.99)	0.65 (0.53-0.74)
**HFP alone**	8.90 (7.82-9.89)		0.62 (0.57-0.67)	0.37 (0.32-0.42)
**HXP-HX**	22.18 (20.83-24.44)	<.0001	0.96 (0.94-0.98)	0.84 (0.80-0.87)
**HXP-H**	20.14 (18.00-22.31)		0.96 (0.93-0.98)	0.79 (0.74-0.83)
**HXP alone**	9.10 (8.35-9.66)		0.68 (0.65-0.71)	0.37 (0.34-0.40)

Abbreviations: HFP, trastuzumab/5-fluorouracil/cisplatin; HFP-H, trastuzumab/5-fluorouracil/cisplatin followed by trastuzumab maintenance; HXP, trastuzumab/capecitabine/cisplatin; HXP-H, trastuzumab/capecitabine/cisplatin followed by trastuzumab maintenance; HXP-HX, trastuzumab/capecitabine/cisplatin followed by trastuzumab/capecitabine maintenance; OS, overall survival.

The same trend was confirmed using multivariable analysis. Compared to the HXP group, the HFP group showed a significantly poorer TST (hazard ratio [HR] 1.61, 95% CI, 1.40-1.85, *P *< .0001) and OS (HR 1.49, 95% CI, 1.33-1.67, *P *< .0001, Table 4). In the HFP group, compared to the HFP-H group, the HFP alone showed a significantly poorer TST (HR 3.51, 95% CI, 2.50-4.94, *P *< .0001) and OS (HR 2.18, 95% CI, 1.63-2.90, *P *< .0001, Table 4). In the HXP group, compared to the HXP-HX group, HXP-H showed a significantly poorer TST (HR 1.31, 95% CI, 1.09-1.56, *P *= .0038) and OS (HR 1.23, 95% CI, 1.04-1.46, *P *= .019), and HXP alone showed a significantly poorer TST (HR 4.74, 95% CI, 4.08-5.51, *P *< .0001) and OS (HR 3.01, 95% CI, 2.62-3.45, *P *< .0001, Table 4).

**Table 4:** Hazard ratio and 95% confidence intervals of time to subsequent treatment and overall survival from univariable and multivariable Cox proportional hazard regression model.

Outcome	Regimen	Univariable		Multivariable^a^	
		Unadjusted HR (95% CI)	*P*	Adjusted HR (95% CI)	*P*
**Time to subsequent treatment**	**HXP**	**Ref.**		**Ref.**	
	HFP	1.61 (1.40-1.84)	.0001	1.61 (1.40-1.85)	<.0001
	HFP-H	Ref.		Ref.	
	HFP	3.46 (2.48-4.82)	<.0001	3.51 (2.50-4.94)	<.0001
	HXP-HX	Ref.		Ref.	
	HXP-H	1.31 (1.09-1.57)	.0033	1.31 (1.09-1.56)	.0038
	HXP	4.69 (4.04-5.45)	<.0001	4.74 (4.08-5.51)	<.0001
**Overall survival**	HXP	Ref.		Ref.	
	HFP	1.50 (1.34-1.68)	<.0001	1.49 (1.33-1.67)	<.0001
	HFP-H	Ref.			
	HFP	1.96 (1.49-2.58)	<.0001	2.18 (1.63-2.90)	<.0001
	HXP-HX	Ref.		Ref.	
	HXP-H	1.22 (1.03-1.45)	.0234	1.23 (1.04-1.46)	.019
	HXP	3.00 (2.61-3.43)	<.0001	3.01 (2.62-3.45)	<.0001

Abbreviations: HFP, trastuzumab/5-fluorouracil/cisplatin; HFP-H, trastuzumab/5-fluorouracil/cisplatin followed by trastuzumab maintenance; HR, hazard ratio; HXP, trastuzumab/capecitabine/cisplatin; HXP-H, trastuzumab/capecitabine/cisplatin followed by trastuzumab maintenance; HXP-HX, trastuzumab/capecitabine/cisplatin followed by trastuzumab/capecitabine maintenance.

a Adjusted by patient’s age, sex, diabetes mellitus, hypertension, chronic ischemic heart disease, chronic kidney disease, cirrhosis of liver, chronic obstructive pulmonary disease, history of cancer.

### Second-line treatment and treatment outcomes

The types of second-line chemotherapeutic agents used for both the HFP and HXP groups are summarized in Table S1, and the regimen with ramucirumab plus paclitaxel was most common. The median time to second subsequent treatment (TST2) for the HFP and HXP groups showed no significant difference (HFP: 5.22 months, 95% CI, 4.07-5.88/HXP: 5.22 months, 95% CI, 4.80-5.75, *P *= .7498, Table S2). This trend was consistent with the subgroup analyses. Comparing the HFP-H and HFP alone groups, median TST2 was not significantly different (HFP-H: 6.64 months, 95% CI, 3.09-9.86/HFP alone: 4.99 months, 95% CI, 4.01-5.62, *P *= .7369, Table S2). Among the HXP-HX, HXP-H, and HXP alone groups, median TST2 also showed no significant difference (HXP-HX: 5.95 months, 95% CI, 4.83-6.74; HXP-H: 5.75 months, 95% CI, 4.60-6.57; HXP alone: 4.80 months, 95% CI, 4.14-5.42, *P *= .3532, Table S2).

## Discussion

Gastric cancer is more prevalent in East Asians and is one of the most common causes of cancer-related mortality worldwide.^16–18^ Since the publication of the ToGA trial results, trastuzumab-based chemotherapy has become the first-line standard treatment for patients with HER2-positive locally advanced unresectable or metastatic GC.^8^ Currently, trastuzumab-based chemotherapy has been reimbursable in the Republic of Korea, allowing for an analysis of real-world treatment outcomes through big data analysis.

In the present study, patients treated with HXP showed better prognosis in both TST and OS than those treated with HFP. Previous randomized trials, including the REAL-2 and ML17032 studies, also reported modest survival advantages of capecitabine-based regimens over 5-FU–based regimens in advanced esophagogastric cancer, which supports the clinical plausibility of our findings.^19^^,^^20^ However, it is challenging to conclude definitively that HXP is superior to HFP based solely on these results. Due to the structural limitations of the HIRA data, we were unable to assess the performance status of the patients.^21^ It is possible that a higher proportion of patients with poorer performance status, recognized as an adverse prognostic factor, was treated with HFP,^22–24^ because capecitabine may cause more side effects than 5-FU, such as hand-foot syndrome.^20^^,^^25^ Additionally, the HFP-H, HXP-H, and HXP-HX groups demonstrated a better prognosis than that of the HFP or HXP groups. This outcome may be attributable to the retrospective nature of this study, as it likely included a substantial number of patients who experienced disease progression before initiating H or HX maintenance therapy, leading to poorer treatment outcomes.

In this study, we mainly focused on the significantly better outcomes observed in patients with HXP-HX than in those treated with HXP-H. In the ToGA trial, the treatment protocol was designed as 6 cycles of triplet therapy (ie, HFP or HXP), followed by trastuzumab maintenance alone.^6^ In real-world practice in Korea, trastuzumab is reimbursed for HER2-positive locally advanced unresectable or metastatic GC; however, the continuation of capecitabine beyond 6 cycles as maintenance therapy is not strictly mandated by reimbursement policy and is generally determined at the physician’s discretion. Therefore, some physicians prescribe capecitabine during trastuzumab maintenance based on individual clinical judgment.^12^^,^^13^^,^^26^^,^^27^ A study suggests that adding a mono-chemo agent to maintenance therapy yields better outcomes; however, this is limited as it was conducted as an observational study with a small number of patients.^13^ The finding that patients who received maintenance therapy with both capecitabine and trastuzumab demonstrated TST and OS benefits in a real-world big data analysis is an encouraging result that could potentially influence treatment strategies for patients with GC.

Additionally, there were no significant differences in median TST2 according to second-line treatment among the groups. Considering that studies proving survival benefits of third-line treatment for patients with GC are limited and confined to marginal benefits,^28–30^ TST differences in first-line treatment likely had a substantial impact on OS outcomes. This result further supports the potential positive effects of the HXP-HX regimen.

Despite the recent findings from the KEYNOTE-811 study, which demonstrated improved outcomes by combining pembrolizumab with the ToGA regimen in patients with HER2-positive GC and a PD-L1 combined positive score of 1 or higher,^31–33^ our findings should be interpreted with caution in the context of the current immunotherapy era. As this study was conducted using data prior to the adoption of PD-1 inhibitors, caution is warranted for direct associations between our findings and the KEYNOTE-811 regimen.

Nevertheless, our results suggest that the addition of capecitabine during maintenance following HXP may confer survival benefit. These findings may provide a rationale for future investigation into capecitabine-containing maintenance strategies in combination with pembrolizumab and trastuzumab. However, given that this study was conducted prior to the incorporation of PD-1 inhibitors, the role of HXP-HX in combination with pembrolizumab should be considered hypothesis generating and requires prospective validation. Further prospective clinical trials or real-world studies incorporating PD-1 inhibitors are needed to determine the optimal maintenance approach and duration of chemotherapy in the immunotherapy era.

This study had several limitations. First, this was a retrospective study, making it challenging to generalize the results. Second, the exact dates of disease progression and death could not be identified because of the nature of the HIRA data,^21^ so these events were operationally defined in this study. As a result, death was defined as the absence of medical records or drug prescriptions for more than 6 months, which may have misclassified surviving patients as deceased. However, because this was a big data analysis and the survival outcomes of this study were comparable to those of the ToGA trial,^8^ it is unlikely that this factor significantly affected the study results. Although this operational definition may misclassify a small proportion of patients who were lost to follow-up or received care outside the Korean national health insurance (NHI) system, the impact is likely limited in the context of the NHI system, where approximately 97% of the Korean population is enrolled.^21^ Given the near-complete capture of healthcare utilization under the NHI system, prolonged absence of claims for 6 months in patients with advanced cancer is highly unlikely to reflect continued survival. This interpretation is further supported by a published validation study of the operational definition of mortality using HIRA data,^34^ which confirmed that the absence of claims for ≥150 days most accurately identified true death in a seriously ill population. However, this approach may be subject to informative censoring, as patients who discontinue healthcare utilization because of end-of-life care outside the NHI system may be more likely to be misclassified. For example, patients transitioning to noncovered hospice care or pursuing alternative treatments could be incorrectly categorized as deceased.^34^ Nonetheless, the prior validation study demonstrated high accuracy of this operational definition, suggesting that the magnitude of such bias is likely to be limited.^34^ Third, the HIRA data did not include information on performance status, so there may have been differences in performance status between treatment groups. Because performance status is an important prognostic factor and may influence treatment selection, patients with poorer performance status may have been more likely to receive HFP rather than HXP, potentially introducing healthy user bias. This possibility is also supported by the baseline characteristics observed in the present study, where the HFP cohort included a higher proportion of 70 or older patients and patients with CKD. However, we attempted to mitigate this limitation by adjusting for available clinical variables associated with general health status, including age and comorbidity profiles such as CKD. Nevertheless, residual confounding related to unmeasured functional status cannot be completely excluded. In particular, the potential for healthy user bias, whereby fitter patients may have been preferentially selected for HXP-based treatment, should be carefully considered when interpreting the observed survival differences. Fourth, this study has the potential for immortal time bias in the maintenance analysis. Patients who received maintenance therapy were required to survive through the induction phase, which may have inherently selected for individuals with more favorable early disease control. However, for our primary comparison of interest, HXP-HX vs HXP-H, immortal time bias is less likely to be a major concern because both groups completed initial HXP treatment and entered the maintenance phase under comparable conditions. Additionally, the Kaplan-Meier curves demonstrated comparable early survival between HXP-HX and HXP-H, with divergence emerging gradually during follow-up rather than immediately at time zero. Furthermore, the 6-month OS rates were nearly identical between the two groups, with separation becoming more apparent at 12 months. This temporal pattern suggests that the observed survival difference may not be solely attributable to immortal time bias. Nevertheless, residual confounding and selection bias cannot be fully excluded given the retrospective nature of this study, and the observed 22.18-month OS in the HXP-HX group compared to the 9.10-month OS in the HXP alone group cannot be interpreted as a purely causal effect due to this structural bias. Fifth, the HIRA database does not provide detailed information on treatment-related adverse events or toxicity grades. Therefore, we were unable to assess the safety profile or real-world tolerability of the HXP-HX regimen, including adverse events such as hand-foot syndrome. This limitation should be considered when interpreting the feasibility of prolonged capecitabine maintenance in clinical practice. Nevertheless, the difference in outcomes between the HXP-HX and HXP-H groups is noteworthy, and it is encouraging that adding capecitabine to trastuzumab demonstrated a survival benefit. Based on the results of this study, HXP-HX may be considered a clinically relevant maintenance strategy in selected patients with locally advanced unresectable or metastatic HER2-positive GC who tolerate capecitabine, although prospective validation is warranted.

## Conclusions

Trastuzumab/capecitabine/cisplatin followed by trastuzumab/capecitabine maintenance demonstrated the best survival outcomes for patients with locally advanced or metastatic HER2-positive GC based on a real-world big data analysis of the ToGA regimen. Therefore, trastuzumab plus capecitabine maintenance may be considered over trastuzumab-only maintenance following HXP in selected patients, although prospective validation is required. In the immunotherapy era for HER2-positive GC, the potential role of capecitabine maintenance in combination strategies should be interpreted cautiously and requires further investigation.

## Supplementary Material

oyag196_Supplementary_Data

## Data Availability

The datasets generated and/or analyzed during the current study are not publicly available due to the confidentiality of patient data, but are available from the corresponding author on reasonable request.
