# Supplementary material for: Treatment outcomes of trastuzumab-based chemotherapy in patients with HER2-positive gastric cancer: a nationwide retrospective cohort study
Source: Oncologist. 2026 May 20;31(7):oyag196. doi: 10.1093/oncolo/oyag196 (PMC13326748; doi:10.1093/oncolo/oyag196)
Supplement: oyag196_Supplementary_Data [file oyag196_supplementary_data.docx]

Supplementary Table S1. Second-line treatment according to the first-line regimen

| **First-line regimen** | **Rank** | **Second-line treatment** | **N** |
| --- | --- | --- | --- |
| HFP | 1 | ramucirumab plus paclitaxel | 54 |
|  | 2 | FOLFIRI | 31 |
|  | 3 | paclitaxel | 21 |
|  | 4 | fluorouracil plus irinotecan | 18 |
|  | 5 | FOLFOX | 16 |
|  | 6 | docetaxel | 15 |
|  | 7 | fluorouracil plus oxaliplatin | 14 |
|  | 8 | docetaxel plus cisplatin | 10 |
|  | 9 | irinotecan | 10 |
| HXP | 1 | ramucirumab plus paclitaxel | 294 |
|  | 2 | paclitaxel | 204 |
|  | 3 | FOLFIRI | 114 |
|  | 4 | fluorouracil plus irinotecan | 91 |
|  | 5 | irinotecan | 75 |
|  | 6 | FOLFOX | 68 |
|  | 7 | docetaxel | 57 |
|  | 8 | fluorouracil plus oxaliplatin | 42 |
|  | 9 | docetaxel plus cisplatin | 17 |
|  | 10 | tegafur/gimeracil/oteracil | 13 |

HFP, trastuzumab/5-fluorouracil/cisplatin; HXP, trastuzumab/capecitabine/cisplatin; FOLFIRI, 5-fluorouracil/leucovorin/irinotecan; FOLFOX, 5-fluorouracil/leucovorin/oxaliplatin.

Supplementary Table S2. Time to second subsequent treatment according to the chemotherapeutic regimen

| **Starting regimen** | **N** | **Median TST2 (95% CI), months** | **Log-rank *P* value** | **6-month second subsequent-treatment-free rate (95% CI)** | **12-month second subsequent-treatment-free rate (95% CI)** |
| --- | --- | --- | --- | --- | --- |
| HFP | 262 | 5.22 (4.07–5.88) | 0.7498 | 0.41 (0.32–0.49) | 0.16 (0.08–0.25) |
| HXP | 1,138 | 5.22 (4.80–5.75) |  | 0.44 (0.40–0.48) | 0.17 (0.14–0.21) |
| HFP-H | 45 | 6.64 (3.09–9.86) | 0.7369 | 0.53 (0.34–0.69) | 0.08 (0.01–0.28) |
| HFP alone | 217 | 4.99 (4.01–5.62) |  | 0.37 (0.27–0.47) | 0.19 (0.10–0.30) |
| HXP-HX | 265 | 5.95 (4.83–6.74) | 0.3532 | 0.48 (0.40–0.56) | 0.17 (0.11–0.25) |
| HXP-H | 216 | 5.75 (4.60–6.57) |  | 0.48 (0.40–0.56) | 0.17 (0.10–0.24) |
| HXP alone | 657 | 4.80 (4.14–5.42) |  | 0.40 (0.34–0.45) | 0.18 (0.13–0.24) |

TST2, time to second subsequent treatment; CI, confidence interval; HFP, trastuzumab/5-fluorouracil/cisplatin; HXP, trastuzumab/capecitabine/cisplatin; HFP-H, trastuzumab/5-fluorouracil/cisplatin followed by trastuzumab maintenance; HXP-HX, trastuzumab/capecitabine/cisplatin followed by trastuzumab/capecitabine maintenance; HXP-H, trastuzumab/capecitabine/cisplatin followed by trastuzumab maintenance.
